# Factors influencing the utilization of doctoral research findings at a university in KwaZulu-Natal, South Africa: Views of academic leaders

**DOI:** 10.1371/journal.pone.0290651

**Published:** 2023-08-31

**Authors:** Florence Upenyu Damba, Ntombifikile Gloria Mtshali, Moses John Chimbari

**Affiliations:** 1 School of Nursing and Public Health, College of Health Sciences, Howard College, University of KwaZulu-Natal, Berea, Durban, South Africa; 2 Great Zimbabwe University, Masvingo, Zimbabwe; Lorestan University of Medical Sciences, ISLAMIC REPUBLIC OF IRAN

## Abstract

**Background:**

Translation of health research findings into policy remains a challenge in sub-Saharan Africa. Factors influencing health research utilization are poorly described in literature. Therefore, identifying factors that influence the utilization of research findings for policy formulation is essential to facilitate implementation of evidence-based interventions. The purpose of this study was to explore the views of academic leaders as to why doctoral research is not adequately used in policymaking.

**Methods:**

In-depth interviews were held with purposively selected key informants from the College of Health Sciences. An open-ended interview guide aimed at exploring college leadership views on factors influencing utilization of PhD generated knowledge into policy was used. Data was analysed thematically using NVivo 12 software. Thematic analysis was used to generate themes around the factors influencing utilization of doctoral research into policy.

**Results:**

Factors such as inaccessibility of research results, lack of funding, poor quality of research, lack of continuity in translating research into policy, lack of timeliness of research results and lack of collaboration between researchers and policymakers hindered the utilization of PhD generated knowledge. Participants recommended engagement with the Department of Health/policymakers, collaboration with Department of Health/policymakers, increasing enrolment of South African citizens into PhD program, making final research products available to Department of Health/policymakers, and provision of funding for dissemination of research results.

**Conclusion:**

The study demonstrated that final doctoral research results are mainly disseminated through journal articles and theses. Participants cited inaccessibility of research findings, lack of funding and poor-quality research as the most common factors hindering utilization of doctoral research findings. The study also recommended availing adequate funding for dissemination of research results, collaboration between researchers and policymakers, facilitation of policymaker-researcher engagement to find best ways of using research findings to influence policy and making final research products accessible to policymakers. Further research to gain the perspective of policymakers as to why doctoral research is not adequately used in policy formulation is recommended.

## Background

Currently there is a global recognition that evidence-informed health policies are essential to achieve continued improvement in health outcomes [1]. The Beijing, Montreux, and Bamako calls to action have emphasized the importance of using health research evidence in the development of health policies [2]. There has been increased pressure on universities to produce relevant and impactful research, engage with non-academic stakeholders who are looking for answers to their challenges and to translate their results and outcomes of research into policy [3]. Despite these calls and the abundance health research findings, health policies in the African region are sub optimally informed by evidence [4]. The suboptimal research use in African countries including South Africa has been mainly attributable to inadequacies in funding, limited access to research evidence, limited capacity to interpret research evidence, the exclusion of policymakers from setting the research agenda [5–8]. This has prompted researchers to investigate the factors that influence the utilization of research findings for policy formulation. Understanding the factors that inhibit or promote utilization of research results is necessary.

Research to understand the factors that influence utilization of research findings into policy has been conducted in other countries [9–11, 13]. A few studies have been conducted in low- and middle- income countries (LMICs) [11, 13] therefore limited knowledge exists on factors that influence the utilization of research findings into policy in (LMICs and South Africa in particular. This study represents academics’ perceptions of the factors that influence utilization of research findings into policy as has been explored by Ion et al. [9]. The study did not take into account policymakers’ perceptions of these issues as has been explored elsewhere [12, 13].

Against this background, we explored the views of academics on the factors that influence utilization of doctoral research findings and their recommendations on increasing utilization of the knowledge. The following research question was addressed “What factors contribute to utilization of doctoral research conducted in the School of Nursing and Public Health by policymakers?”

## Methods

### Study area

The study was conducted in KwaZulu-Natal (KZN) province and data was collected at the University of KwaZulu-Natal (UKZN) in the College of Health Sciences (CHS), School of Nursing and Public Health (SNPH). We chose UKZN because it is one of the largest universities with a large public health unit that will give us key information.

### Study design

A case study design applying the qualitative method approach was applied in this study. The School of Nursing and Public Health in the College of Health Sciences was treated as the case. The case study design was adopted because it allows the researcher to investigate a topic in its real- life context [14]. The study was qualitative and employed a structured interview guide to conduct in-depth interviews for data collection. Data was collected between December 2020 to June 2021. In-depth interviews were used because they can reveal new information, uncover hidden practices, and provide insight into the topic [15, 16] which is imperative in understanding factors that influence the utilization of PhD generated knowledge/doctoral research findings for policy formulation. In-depth interviews were also used to seek insights of those who had experienced or were experiencing the phenomenon [17, 18]. The research question addressed by the study was “What factors contribute to utilization of doctoral research findings in the SNPH, UKZN by policymakers?”

#### Study population and sampling

Purposive sampling as defined by Teddlie, and Yu [19] was adopted to recruit College Leadership personnel to participate in the study. We defined College Leadership as parties holding leadership positions in the College of Heath Sciences (CHS). The following key informants were purposively selected to participate in the study: the Deputy Vice Chancellor of the College of Health Sciences (1), the Dean of Research 2013–2017 (1), the Dean of Research for 2017–2021 (1), the Dean and Head of School of Nursing and Public Health (1), the Academic Leader for Research (1), the PhD Coordinator in the School of Nursing and Public Health (1), Head of Departments for the 9 Disciplines in the School of Nursing and Public Health (9). Views of leadership are important as interventions to improve the situation require buy-in of leadership. We accept that such selection can have limitations on the generalizability of the study findings. However, the study provides good insights on leadership views and perceptions regarding utilization of doctoral research for policy formulation. Out of the targeted sixteen College Leadership personnel only ten (62.5%) were interviewed. Six (37.5%) were unavailable.

#### Data collection methods and process

Individual in-depth interviews were conducted with College Leadership personnel who served as key informants [20]. A structured interview guide which included open-ended questions informed by literature review and the objective of the study was used [21]. The interview guide was designed to explore key informants’ views on the factors that influence the utilization of doctoral research findings from the SNPH, UKZN by policymakers. The researchers focused on ensuring the interview questions were constructed to answer the research question. Probing questions were used to explore the participants’ views that required further clarifications [22]. Invitations and appointments were made with participants via email. Because of COVID-19 restrictions 9 of the interviews were conducted through zoom while one was conducted face-to-face in the participant’s office. Of the nine interviews that were conducted through zoom, one was conducted by a research assistant because there was conflict of interest on the part of the researcher. The interviewee is my spouse. He had to be interviewed because he was the only one in that position during the period under study and there was no deputy. The interviews lasted 40 to 60 minutes. All ten interviews were recorded either on zoom or voice recorder with permission from the participants. Data saturation was reached when the participants were no longer giving new information and the study objective was addressed [23]. We selected quotes from the interviews that were appropriate for the themes that we reported on [24]. Interviewees were assigned unique participant identities in the order in which the interviews were conducted, for example, interviewee 1 is Participant 1 (P1) and so on to protect participant confidentiality. The researcher stored interview transcripts in a secure place as back-up.

#### Analysis

The reflexive thematic analysis method approach was followed during the data analysis process [25–27]. All recorded interviews were transcribed verbatim in a Microsoft word document by the researcher (FUD) and research assistant (CM). All transcripts were validated by the researchers. FUD and CM listened and familiarized themselves with the collected data by using the audio recordings and transcripts. FUD and CM carefully read the transcripts line by line while taking notes of paraphrases or labelling ‘codes’ that were relevant and important. Notes of key ideas and repeated themes were highlighted and taken. The researchers did not code every piece of text. They used open coding since they developed and modified the codes as they worked through the coding process. The main aim of the coding was to categorize all collected data. Coding, searching, and retrieving of data was facilitated by using NVivo software (version 12, QSR International Pty, Ltd, Melbourne, Australia). Codes were then grouped into categories. The categories formed a working analytical framework which was used to perform the indexing of all transcripts using categories and codes. NVivo 12 software was used to generate framework matrices. The characteristics of and the differences between the data were identified. Mapping and interpretation of the connections between categories to relationships and/or causality was done.

#### Rigour/quality/validity and reliability

Prior to conducting the in-depth interviews, the interview guide was pilot tested on College Leadership personnel who did not take part in the study to check the validity and credibility of the tool. The pilot test was used to improve precision, reliability, validity of data, identifying problems/omissions, and assessing time needed to complete the interview. The interview guide was also pilot tested to ascertain if participants interpreted the meaning of the questions as intended. The research instrument was reviewed by experts in the field of research and unclear questions were revised based on the reviewers’ comments [28]. Authenticity was achieved by using direct quotes from different participants [29]. Verbatim quotes enhanced the confirmability of the findings [30].

## Ethics approval

The study was approved by the research ethics committee of the university Biomedical Research Ethics Committee (reference number BREC/00001384/2020) and the Kwa-Zulu-Natal Provincial Department of Health (reference number KZ_202008_030). All research was performed in accordance with the ethical standards of institutional research committee applicable when human participants are involved. Written informed consent was obtained from all the participants in the study.

## Results

Two major themes were generated during the interviews: factors hindering utilization of doctoral research findings into policy (inaccessibility of research results, funding, quality of research and gaps to translate research) and factors promoting utilization of doctoral research findings into policy (engagement with DOH/policymakers, collaboration with DOH/policymakers, increasing enrolment of South African citizens into PhD program, making final research products available to DOH/policymakers and provision of funding for dissemination of research results).

### Theme 1: Factors hindering utilization of doctoral research findings into policy

#### Category 1: Inaccessibility of research results

Participants highlighted different factors associated with inaccessibility of research findings to end users (1, 2, 3, 5, 7 and 8). P1, 3 and 7 were concerned about the formats in which research is packaged and disseminated to policymakers. Researchers’ limited commitment to disseminate research results to policymakers was expressed by P2. P5 raised concern about the researchers’ poor appreciation of the importance of giving feedback.

Participants argued that research remains inaccessible to policymakers mainly because researchers are not packaging evidence in formats that can easily be used by policymakers, for example, theses are long and time consuming. Participants argued that policymakers do not have time to sift through a thesis for information, so researchers need to simplify it. The views of the participants are highlighted in the preceding quotes,

“*I always hear students saying that I will give my thesis to the policymakers*. *There is no policymaker who has time to be reading the thesis”*.

Interviewee 1.

*“To say that we send our theses to the library is not enough*, *we need to simplify them”*.

Interviewee 7

The participants also reflected on the manner and format in which peer- reviewed journal articles are presented to DOH.

*“The manner and format in which it must be presented to DOH to be operationalized requires a different approach to that required in a peer review journal”*.

Interviewee 3

Participants revealed a lack of commitment to disseminate research findings by researchers as well as lack of commitment to utilize research results by DOH. They noted that even though the approval letter from DOH states that the researcher will be expected to provide feedback on findings to the district or facility, DOH often complains that they do not get feedback from students.

*“We should take responsibility all of us*. *It is an obligation for a student to give feedback and if you read the ethics approval that comes from DOH it does talk about you giving them back results*, *but we do not do that*. *On the other hand*, *DOH are supposed to demand feedback”*.

Interviewee 2

*“I sit on the Research Ethics Board of the DOH where they often complain that they do not get reports back from students who have conducted research*. *Only a few researchers report their findings to DOH”*.

Interviewee 8

#### Category 2: Funding

Some of the participants pointed out that students do not get adequate funding to support high impact research. Interviewee 7 indicated that the money allocated for research is not adequate to cover the dissemination of research results to policymakers.

*“If researchers want to give feedback to DOH*, *the community and stakeholders it might require additional resources*. *The school only gives students R40 000 per year which is a total of R80 000 in the 2 years of their research*. *R80 000 is not enough so giving feedback becomes a luxury to them”*.

*Interviewee 7*

Participants concurred that due to inadequate funding students end up using convenient sampling instead of random sampling as a way of saving money.

*“Some of the projects are basically done in a way that should lead to saving money*. *l am having a study right now where we did random sampling of the whole of KZN*. *Sometimes the schools that we want to look at you may have to travel 200km to go the next school and another one will be 100km away so that’s a lot of money to spend to go to that school”*.

*Interviewee 7*

*We find that you have a student who is capable and is a good quality student but for the type of research they would like to do*, *there is just not enough funds and then this impact on the work they are able to do”*.

Interviewee 4

#### Category 3: Quality of research

Participants revealed several factors that compromise the quality of PhD research, and they are echoed in the quotes below:

*“I find that in terms of selection*, *supervisors do not select students well”*.

Interviewee 1

*“Sometimes supervisors complain of students who cannot write and who do not know how to write in an academic manner*. *Sometimes supervisors come across students who do not know what they want to do*, *and they have not even researched around the topic they want to work on”*.

Interviewee 2

*“There is also a lot of pressure on supervisors to take on large numbers of students and to graduate large numbers of students and this tends to put a lot of pressure on supervisors”*.

*Interviewee 3*

*“We have too many students against too few qualified supervisors*. *That compromises our quality”*.

*Interviewee 4*

*“There is lack of experienced supervisors who can supervise”*.

Interviewee 5

*“Some of the supervisors just finished their PhD and are now supervising PhDs so quality is compromised”*.

*Interviewee 7*

#### Category 4: Limited knowledge on translating research into policy

Participants agreed that both researchers and supervisors lack training in policy processes.

*“A lot of academics are not trained in policy processes especially because it involves politics*.*”*

Interviewee 5

“*Academia not trained in policy processes*. *If we were better trained in policy processes generally then we could facilitate research that will influence policy”*.

Interviewee 9

#### Category 5: The duration of PhD studies

Participants felt that doctoral research results are often not timely due to the length of time taken to complete a PhD program which is normally 2 to 4 years.

*“We tend to take a long time to answer one question*. *Policymakers usually want an answer right away*. *They don’t necessarily want to spend four years waiting for an answer when they are dealing with a problem that is in front of them”*.

Interviewee 5

#### Category 6: Lack of incentive to translate research

Lack of incentives for researchers to influence policy was mentioned as another factor influencing utilization of doctoral research findings into policy.

“*There are no incentives for academics to influence policy*. *It now depends on people who are interested in influencing policy*. *If there were incentives*, *then academics would commit to engaging in policy*.*”*.

Interviewee 5

#### Category 7: Lack of continuity to translate research

Another factor said to be affecting the translation of doctoral research into policy is the lack of continuity in translating research into policy. Most doctoral students in CHS are not South African citizens, but their studies will be based on South Africa. Undoubtedly, when they complete their studies, they will go back to their countries without disseminating their research findings.

*“Most of the graduates from doctoral studies are not South African citizens now that poses problems*. *Most students will finish their PhD and go back to their countries so in terms of continuation and interaction with the DOH and relevant authorities here that creates a gap”*.

*Interviewee 6*

### Theme 2: Factors promoting utilization of doctoral research findings into policy

#### Category 1: Making final research products accessible to DOH/policymakers

A very important factor the participants highlighted was the need to make research results accessible to DOH and policymakers through DOH research day.

“*Once their research is done*, *they need to showcase their work*. *The DOH have a National Research Day every year and they invite us*, *so we need to go there in numbers and present our research findings”*.

*Interviewee 2*

“……*she submitted an abstract where she was able to present her findings to the department and in that meeting even the head of department and the MEC were present listening to the research that is being done in the department*. *I think that more of that can really help”*

Interviewee 5

One of the participants suggested that the students can also take advantage of *the* College of Health Sciences (CHS) symposium to present their research findings to DOH.

“*At the College of Health Sciences Symposium*, *instead of inviting people from DOH to officiate they can instead invite representatives from all the 11 districts in KZN*. *To make sure that they come they can give them tasks like chairing the session or doing the adjudication to find the best student*, *or they can even create something that says we want to give an award for research with very practical policy implications and they can find a funder to fund that*. *The people who would be deciding on that are your district managers and every year they would look forward to it to say we want to see the best thesis or the best research paper that came out”*.

Interviewee 7

Participants established that doctoral students disseminate their research results to policymakers through peer-reviewed journal articles which policymakers are likely to cite for policy formulation.

“*If research is not translated into a peer reviewed publication*, *it is not easy to use for policy processes because you cannot really cite it*. *But once we have done that or taken it through those processes it is a lot easier to use”*.

Interviewee 5

Participants recommended researchers to produce policy briefs that policymakers can use with ease during policy formulation.

*“There is no clear pathway on how the findings should be flowing back to them*. *For me*, *a clear pathway would be policy briefs because they do not have time to read long thick theses*.*”*

Interviewee 2

*“Policy is easier to influence when research results are presented in a way that is synthesized for policy purposes such as policy briefs”*.

Interviewee 5

*“Making evidence available publicly and talking about it publicly makes a lot of difference*. *Even though speaking in the media about your research does not change policy*, *it makes policymakers aware of what you are saying because you articulate your evidence differently when you are speaking in public media compared to when you are speaking in a scientific conference”*.

Interviewee 5

Social media platforms such as Twitter and LinkedIn were cited as effective channels that doctoral students can utilize to disseminate their research results to policymakers.

*“Use of social media platforms such as Twitter and LinkedIn since one does not need to pay to present*. *They are free and one gets instant feedback”*.

Interviewee 2

Another participant suggested that a policy paper could also facilitate translation of research into policy.

“*We need to make sure that our students do write a policy paper which looks at how their research can translate into policy*. *The students need to make sure that their discussion chapter has some element of a policy chapter*”.

Interviewee 3

#### Category 2: Provision of funding for dissemination of research results to DOH/policymakers

Availing funding for the dissemination of research findings was also highlighted as a factor that promotes utilization of research results.

*“When scholarships are being awarded the template that is there for spending the money does not emphasise dissemination yet your ethics approval does talk of the dissemination so l would say that perhaps when scholarships are being given particularly internally*, *a certain amount be given for dissemination*.*”*

*Interviewee 7*, CHS Leadership

#### Category 3: Engagement with DOH/policymakers

Participants also highlighted the need for researchers to engage with policymakers right from the proposal development stage to give them influence over the work.

“*Right at proposal development*, *there must be engagement with DOH*. *The proposals that are generated should always highlight policy implications at the time that they are developed with researchers stating the real problem that they want to solve”*.

Interviewee 7

#### Category 4: Collaboration between researchers and policymakers

Collaboration between researchers and policymakers through joint forums and appointments was also said to promote utilization.

“*We have joint forums like the DOH/UKZN joint forum where we could exploit the idea of sharing research which influences policy”*.

Interviewee 3

*“Late last year we were informed that our former Dean of our School was appointed to the policy commission of the DOH in the province and l think that is an important development because it provides a mechanism by which research can be translated into policy more directly”*.

Interviewee 4

#### Category 5: Increasing enrolment of South African citizens into PhD programme

Another noteworthy factor highlighted by the participants is the need to increase the enrolment of students from rural communities into the PhD program. Participants believe that students from disadvantaged communities understand the issues affecting them better which will motivate them to carry out relevant research.

*“There is need to encourage more South African citizens especially black South Africans to be enrolled in the doctoral programs because people from rural areas will understand their needs better since the issues relate to their community and in a way*, *it will motivate them to carry out relevant research”*.

Interviewee 6

## Discussion

The study sought to identify factors that influence utilization of doctoral research findings into policy. Two themes were generated from the study; factors that inhibit the utilization of doctoral research findings into policy and factors that promote utilization. Lack of familiarity with effective dissemination strategies, lack of financial and institutional support for dissemination were generated as primary factors that influence the utilization of doctoral research findings for policy formulation. Participants reported that evidence generated from doctoral research is normally communicated through journal publications which may not be accessible to policymakers because they are written in a language that is difficult for policymakers to understand and use for policymaking [31, 32]. They emphasized that packaging of research results must not be a one size fit all but that a different approach must be applied when peer- reviewed journal articles are presented to policymakers [33]. Limited funding was identified as a crucial factor that influences the translation of doctoral research findings into policy. Participants revealed that R40 000 (approximately US$2 700) allocated for research per annum is not adequate to get the student to do the work throughout the pipeline of disseminating the results to implementation [34, 35].

Doctoral health research is underfunded because universities have severely constrained financial budgets coupled with competing demands [36]. Due to inadequate funding students end up compromising the quality of their research outputs. In most cases studies will be done in a way that leads to saving money. Additional funds may be required for students to organize research findings dissemination workshops to give feedback to DOH, the community and relevant stakeholders. Due to the complex nature of the policy environment, researchers will need the capacity not to just produce knowledge but to navigate the complex terrain [34, 37]. Capacity strengthening can be achieved through collaboration between researchers and policymakers [38]. The time taken to complete a PhD program is often too long for policymakers whose window for making a policy is much shorter [39, 40]. This finding aligns with the findings of other studies that found out that a lack of timely research outputs was one of the key factors impeding utilization of research findings into policy [41–45].

The study revealed several factors that compromised the quality of PhD research. These included PhD students who were underprepared for PhD, students researching on topics which are outside their research areas because they did not have a topic on admission to the programme or their research is part of their supervisor’s big project, supervisors lack of experience to meet the demand for doctoral supervision and supervisors burdened with heavy supervision workloads. Some of the supervisors were reported to have just completed their PhD which also compromises the quality of research. These findings are not unique to this study [46–49]. Heavy supervision workloads ultimately limit supervision capacity to provide effective supervision [50–53].

Participants noted that there are no incentives to motivate researchers to contribute to the development of public health policies. Researchers may perceive active involvement in policy processes as very demanding and time-consuming and are therefore more likely to pursue rewarding academic interests that are sometimes disconnected from policy priorities [34]. Furthermore, donor agencies push their agendas to policymakers leading to less promotion for evidence produced by academic researchers [54]. The study also found that there is lack of continuity to disseminate research results to DOH and other stakeholders because most students in the SNPH are not South African citizens therefore when they finish their PhD programme, they go back to their countries without sharing their results with DOH.

A very important factor promoting utilization of doctoral research findings is the format of dissemination of research evidence, which participants noted must be tailored to suit the needs of policymakers [55–57]. Study participants emphasized that researchers ought to use clear language and tailored audience specific products that meet the needs of diverse end users which corroborates previous studies reporting that policymakers are likely to use research evidence for policymaking when the evidence is available and accessible in a timely and user-friendly format [55, 58–60]. They suggested presentation of research findings at conferences such as KZN-DOH research day which is normally attended by the Member of the Executive Council (MEC) whom participants believed would facilitate uptake of research findings. A research day was successfully utilized as a platform for a policy dialogue between researchers and policymakers for improving maternal, new-born and child health in Nigeria [61]. Participants also recommended presenting research findings at the CHS symposium and suggested that instead of inviting people from DOH to officiate the event, they can give them tasks like chairing or doing the adjudication to find the best student and award them for research with very practical policy implications. Importantly, this may inspire students who have not been fully committed or who did not appreciate the importance of disseminating their research outputs to DOH to do so. It was established that doctoral students disseminate their research results to policymakers through peer-reviewed journal articles providing the likelihood of them being cited for policy [62] since they would have gone through the processes of checking for scientific credibility and technical quality [55, 63]. Participants pointed out that dissemination strategies should go beyond making research available through traditional methods such as peer- reviewed journal articles, theses and conferences [62]. They recommended that students use methods such as policy briefs, elevator speeches, speaking on the media, social media platforms such as Twitter and LinkedIn and policy papers. These solutions have also been suggested in other studies [57, 64]. Participants recommended the use of policy briefs because policymakers do not have time to read [65–67]. This is supported by reports from other low-and middle-income countries that have shown that the use of policy briefs to promote evidence-informed policymaking is well received by policymakers [68, 69]. Participants suggested one- page policy briefs which will provide a short description of the findings and specific recommendations with minimal use of technical jargon that can be quickly examined and interpreted [70].

Participants highlighted that speaking on the radio will reach a wide range of audiences as it gives the researchers the opportunity to communicate with policymakers, the public and journalists which can almost be impossible with peer-reviewed journal articles and scientific conferences [71]. They argued that it makes policymakers aware of available evidence and at the same time enabling one to articulate their evidence differently as compared to when one is speaking in a scientific conference. Use of social media was also recommended because it offers convenience in accessing evidence at any given time as well as allowing instant feedback between researchers and policymakers [62]. Participants recommended that when scholarships are being awarded to students internally, a certain amount should be allocated for the dissemination of research findings to policymakers. Participants noted that right at proposal development there must be engagement with policymakers to facilitate effective research uptake by giving stakeholders influence over topic selection, research process and outcome implementation [72–74].

Participants also stated that proposals that are generated should always highlight policy implications and the student should be able to state the real problem they want to solve. This might increase the likelihood that policymakers will remain invested in the work by allowing individual research interests to shine [75, 76]. This is in line with findings from upper- income countries that emphasize early and sustained engagements between researchers and policymakers [72, 73, 77–79]. Participants emphasized that collaboration between researchers and policymakers will create opportunities of sharing research and awareness of policy relevance of research projects, and stimulate interest among policymakers to discuss how the research findings might be translated and used to support policy discussions [80–82]. Examples of such collaborations cited in the interviews were the university having joint forums with DOH such as the DOH/CHS joint health agreement which is currently being negotiated on where they can share research that influences policy. Another interesting collaboration that the participants believed to be an important development was the appointment of the former Dean of the SNPH to policy commission of DOH in the province which participants hope will provide a mechanism by which research can be translated into policy more directly.

## Strengths and limitations

To the researchers’ knowledge this is the first qualitative study to provide an in-depth qualitative understanding of the factors that influence the utilization of doctoral research findings for policy formulation, and it will add value to the limited studies in this area. The use of purposive sampling and the fact that the study was conducted in a single setting and with a smaller number of participants present the main limitation of the study. It limits the potential of generalizing the findings to the entire university, as factors that influence utilization of doctoral research findings in each school may be unique. Our findings should be interpreted cautiously and contextualized. We, however, think that there are good lessons from this case study. Future studies may need to increase the study population to have a larger sampling frame that provides better generalizability to the reference population.

## Conclusion

Engaging policymakers earlier in the projects, maintaining communication during the research process, and presenting relevant findings in a clear, concise manner may empower both researchers and policymakers to improve health policy. It is recommended that the SNPH consistently encourage the provision of research results to policymakers in the same manner they encourage publication of research findings in peer-reviewed journals. We noted that money allocated for research and research findings dissemination is not adequate and there is therefore a strong need to advocate for stronger funding mechanisms to support knowledge production and dissemination.

Students are recommended to take advantage of capacity development workshops which include proposal development, mentorship, manuscript writing and theses writing which are all meant to make sure that at every stage a student does not find themselves isolated. The study established that academics are not trained in policy processes therefore they do not know how to influence translation of research into policy. We therefore propose that researchers avail themselves to participate in policy processes to be politically minded knowing when to produce evidence and how to get it across to the users. It was also suggested that the university gets into portfolio committees of health through the political structures of DOH to facilitate policymaker- researcher engagement in finding best ways of using research findings to influence policy. The study found that since most students in the SNPH are not South African citizens there is lack of continuity in translating research findings into policy. When the students finish their PhD programme, they go back to their countries without sharing their results with DOH. In this regard we recommend increasing the enrolment of SA citizens into PhD programs to facilitate continuation and interaction with DOH and relevant authorities.

## Supporting information

S1 Checklist Standard for Reporting Qualitative Research (SRQR) checklist. (DOCX)

S1 File This file contains the interview guide with College Leadership personnel. (DOCX)
