# Supplementary material for: Factors influencing the utilization of doctoral research findings at a university in KwaZulu-Natal, South Africa: Views of academic leaders
Source: PLoS One. 2023 Aug 31;18(8):e0290651. doi: 10.1371/journal.pone.0290651 (PMC10470883; doi:10.1371/journal.pone.0290651)
Supplement: S1 Checklist — (DOCX) [file pone.0290651.s001.docx]

|  | **Standards for Reporting Qualitative Research (SRQR)*** |  |
| --- | --- | --- |
|  | <http://www.equator-network.org/reporting-guidelines/srqr/> |  |
|  |  | **Page/line no(s).** |
| **Title and abstract** | |  |
|  | **Title** - Concise description of the nature and topic of the study Identifying the study as qualitative or indicating the approach (e.g., ethnography, grounded theory) or data collection methods (e.g., interview, focus group) is recommended | The topic of the manuscript does not concisely describe the nature and topic of the study as qualitative or indicating the approach or data collection methods as recommended. |
|  | **Abstract** - Summary of key elements of the study using the abstract format of the intended publication; typically includes background, purpose, methods, results, and conclusions | Page 1 (Lines 12- 20) and Page 2 (Lines 21- 42). |
|  |  |  |
| **Introduction** | |  |
|  | **Problem formulation** - Description and significance of the problem/phenomenon studied; review of relevant theory and empirical work; problem statement | Page 3 (Lines 46- 64). |
|  | **Purpose or research questio**n - Purpose of the study and specific objectives or questions | Page 3 (Line 67); Page 4 (Lines 68- 69). |
|  |  |  |
| **Methods** | |  |
|  | **Qualitative approach and research paradigm** - Qualitative approach (e.g., ethnography, grounded theory, case study, phenomenology, narrative research) and guiding theory if appropriate; identifying the research paradigm (e.g., postpositivist, constructivist/ interpretivist) is also recommended; rationale** | Page 4 (Lines 78- 81). |
|  | **Researcher characteristics and reflexivity** - Researchers’ characteristics that may influence the research, including personal attributes, qualifications/experience, relationship with participants, assumptions, and/or presuppositions; potential or actual interaction between researchers’ characteristics and the research questions, approach, methods, results, and/or transferability | We do not have a specific section addressing those aspects in our manuscript. However, some of the aspects are mainstreamed in the manuscript. |
|  | **Context** - Setting/site and salient contextual factors; rationale** | Page 4 (Lines 72- 75). |
|  | **Sampling strategy** - How and why research participants, documents, or events were selected; criteria for deciding when no further sampling was necessary (e.g., sampling saturation); rationale** | Page 5 (Lines 92-102). |
|  | **Ethical issues pertaining to human subjects** - Documentation of approval by an appropriate ethics review board and participant consent, or explanation for lack thereof; other confidentiality and data security issues | Page 7 (Lines 151- 156). |
|  | **Data collection methods** - Types of data collected; details of data collection procedures including (as appropriate) start and stop dates of data collection and analysis, iterative process, triangulation of sources/methods, and modification of procedures in response to evolving study findings; rationale** | Page 4 (Lines 81- 88); Page 5 (Lines 104- 114); Page 6 (Lines 115- 139). |
|  | **Data collection instruments and technologies** - Description of instruments (e.g., interview guides, questionnaires) and devices (e.g., audio recorders) used for data collection, if/how the instrument(s) changed over the course of the study | Page 5 (Lines 104- 114); Page 6 (Lines 115- 126). |
|  | **Units of study** - Number and relevant characteristics of participants, documents, or events included in the study; level of participation (could be reported in results) | Page 5 (Lines 92- 102). |
|  | **Data processing** - Methods for processing data prior to and during analysis, including transcription, data entry, data management and security, verification of data integrity, data coding, and anonymization/de-identification of excerpts | Page 6 (Line 118- 121, and 124- 126). |
|  | **Data analysis** - Process by which inferences, themes, etc., were identified and developed, including the researchers involved in data analysis; usually references a specific paradigm or approach; rationale** | Page 6 (Lines 123- 139). |
|  | **Techniques to enhance trustworthiness** - Techniques to enhance trustworthiness and credibility of data analysis (e.g., member checking, audit trail, triangulation); rationale** | Page 7 (Lines 141- 149). |
|  |  |  |
| **Results/findings** | |  |
|  | **Synthesis and interpretation** - Main findings (e.g., interpretations, inferences, and themes); might include development of a theory or model, or integration with prior research or theory | Page 7 (Lines 159- 162); Page 8 (Lines 163- 178, and 1183- 184); Page 9 (Lines 187- 191, and 199- 202); Page 10 (Lines 207- 208, and 216- 218); Page 11 (Lines 232- 233, 238- 240, 244- 246, and 250); Page 12 (Lines 251- 253, and 261- 265); Page 13 (Lines 273- 274, 283- 285, and 290- 291); Page 14 (Lines 302- 303, 306- 307, and 311- 313); Page 15 (Lines 318- 320, 324- 326, and 333- 337); Page 21 (Lines 480- 484); Page 22 (Lines 485- 501). |
|  | **Links to empirical data** - Evidence (e.g., quotes, field notes, text excerpts, photographs) to substantiate analytic findings | Page 8 (Lines 179- 182); Page 9 (Lines 185- 186, 192- 194, 196- 198, and 203-206); Page 10 (Lines 209- 215, and 219- 229); Page 11 (Lines 230- 231, 234- 237, 241- 243, and 247- 249); Page 12 (Lines 256- 258, 259- 262 and 266- 271); Page 13 (Lines 275- 281, 286- 288, and 292- 293); Page 14 (Lines 295- 301, 304- 305, and 308- 310, and 314- 317); Page 15 (Lines 321- 323, 327- 332, and 338- 339); Page 16 (Lines 340- 341). |
|  |  |  |
| **Discussion** | |  |
|  | **Integration with prior work, implications, transferability, and contribution(s) to the field -** Short summary of main findings; explanation of how findings and conclusions connect to, support, elaborate on, or challenge conclusions of earlier scholarship; discussion of scope of application/generalizability; identification of unique contribution(s) to scholarship in a discipline or field | Page 16 (Lines 343- 363); Page 17 (Lines 364- 387); Page 18 (388- 412); Page 19 (Lines 413- 436); Page 20 (Lines 437- 461); Page 21 (Lines 462- 466). |
|  | **Limitations** - Trustworthiness and limitations of findings | Page 21 (Lines 469- 478). |
|  |  |  |
| **Other** | |  |
|  | **Conflicts of interest** - Potential sources of influence or perceived influence on study conduct and conclusions; how these were managed | Page 23 (Line 520). |
|  | **Funding** - Sources of funding and other support; role of funders in data collection, interpretation, and reporting | Cover letter |
|  |  |  |
|  | *The authors created the SRQR by searching the literature to identify guidelines, reporting standards, and critical appraisal criteria for qualitative research; reviewing the reference lists of retrieved sources; and contacting experts to gain feedback. The SRQR aims to improve the transparency of all aspects of qualitative research by providing clear standards for reporting qualitative research. |  |
|  |  |  |
|  | **The rationale should briefly discuss the justification for choosing that theory, approach, method, or technique rather than other options available, the assumptions and limitations implicit in those choices, and how those choices influence study conclusions and transferability. As appropriate, the rationale for several items might be discussed together. |  |
|  |  |  |
|  | **Reference:** |  |
|  | O'Brien BC, Harris IB, Beckman TJ, Reed DA, Cook DA. **Standards for reporting qualitative research: a synthesis of recommendations.** *Academic Medicine*, Vol. 89, No. 9 / Sept 2014  DOI: 10.1097/ACM.0000000000000388 |  |
|  |  |  |
|  |  |  |
