# Supplementary material for: Factors influencing the utilization of doctoral research findings at a university in KwaZulu-Natal, South Africa: Views of academic leaders
Source: PLoS One. 2023 Aug 31;18(8):e0290651. doi: 10.1371/journal.pone.0290651 (PMC10470883; doi:10.1371/journal.pone.0290651)
Supplement: S1 File — (DOCX) [file pone.0290651.s002.docx]

**Interview schedule with College Leadership- College of Health Sciences, University of KwaZulu Natal**

1. Position Held ...............................................................

2. Length of time in current position .................................................................

3. Number of students currently being supervised ............................................

4. What is your highest education level ................................................................?

Good day and thank you for agreeing to be interviewed for my study. I trust you are aware of policy relevance/impact of PhD work and how health systems in South Africa can benefit greatly from knowledge generated by universities through PhD work. I am interested to hear of your knowledge and experience of how research done at PhD level can influence policy formulation.

1. What do you think should be done by researchers or PhD students for their research findings to be visible and applied during policy formulation?
2. What do you think are the challenges, if any, of having doctoral research findings from your college translated into policy?
3. Please describe the quality of research generated in your school.
4. Do you think it is regarded as addressing current health issues faced by the health sector?
5. In your opinion, what are the barriers to translation of research findings into policy?
6. Health research is expected to improve health outcomes, do you think research generated in your school is making any contribution to the health system of South Africa?
7. What do you think would result in research generated in your school an attractive option for policymakers and those responsible for policy formulation in the Department of Health?
8. What do you think can facilitate translation of health research findings into policy?
9. Are you aware of any opportunities for the school/college to influence policy through research done by doctoral students?
10. What are the challenges faced by the school/college in terms of quality of research done by PhD students?
